# Unit cost analysis of training and deploying paid community health workers in three rural districts of Tanzania

**DOI:** 10.1186/s12913-016-1476-5

**Published:** 2016-07-08

**Authors:** Kassimu Tani, Amon Exavery, Colin D. Baynes, Senga Pemba, Ahmed Hingora, Fatuma Manzi, James F. Phillips, Almamy Malick Kanté

**Affiliations:** Ifakara Health Institute, P.O. Box 78373, Dar es Salaam, Tanzania; Heilbrunn Department of Population and Family Health, Mailman School of Public Health, Columbia University, 60 Haven Avenue (B2), New York, NY 10032 USA; Tanzanian Training Centre for International Health, P.O. BOX 39, Ifakara, Tanzania

**Keywords:** Community health worker, Training cost, Operation cost, Primary care, Tanzania

## Abstract

**Background:**

Tanzania, like other African countries, faces significant health workforce shortages. With advisory and partnership from Columbia University, the Ifakara Health Institute and the Tanzanian Training Centre for International Health (TTCIH) developed and implemented the Connect Project as a randomized cluster experimental trial of the childhood survival impact of recruiting, training, and deploying of a new cadre of paid community health workers (CHW), named “Wawazesha wa afya ya Jamii” (WAJA). This paper presents an estimation of the cost of training and deploying WAJA in three rural districts of Tanzania.

**Methods:**

Costing data were collected by tracking project activity expenditure records and conducting in-depth interviews of TTCIH staff who have led the training and deployment of WAJA, as well as their counterparts at Public Clinical Training Centres who have responsibility for scaling up the WAJA training program. The trial is registered with the International Standard Randomized Controlled Trial Register number (ISRCTN96819844).

**Results:**

The Connect training cost was US$ 2,489.3 per WAJA, of which 40.1 % was for meals, 20.2 % for accommodation 10.2 % for tuition fees and the remaining 29.5 % for other costs including instruction and training facilities and field allowance. A comparable training program estimated unit cost for scaling-up this training via regional/district clinical training centres would be US$ 833.5 per WAJA. Of this unit cost, 50.3 % would involve the cost of meals, 27.4 % training fees, 13.7 % for field allowances, 9 % for accommodation and medical insurance. The annual running cost of WAJA in a village will cost US$ 1.16 per capita.

**Conclusion:**

Costs estimated by this study are likely to be sustainable on a large scale, particularly if existing regional/district institutions are utilized for this program.

## Background

Tanzania, like many other African countries, is compelled to confront its high burden of disease with a low ratio of population density of health workers (HW) [1, 2]. Human resource shortages rank among the most prominent challenges that must be addressed if health system performance is to be improved [1, 3]. A confluence of factors contribute to the manpower crisis: rapid population growth, health system resource constraints and inadequate worker training [4], emigration of health professionals, or shifting employment from the health sector to other pursuits within the country [5]. In sub-Saharan Africa, these pervasive problems are exacerbated by systems constraints that vary by country, but typically include lack of capacity to absorb and deploy health workers, inadequate investments in pre-service training, shifts to other non-health professions that are motivated by low levels of compensation and by the workforce effects of the high prevalence of HIV/AIDS [4, 6].

The regional toll of these problems is striking. In 2006, the World Health Organization (WHO) estimated that Africa had only 2.3 healthcare workers per 1,000 population compared with 4.3 for Southeast Asia and 24.8 for North America [7]. At 1.35 healthcare workers per 1,000 population and 1 physician per 25,000 population in 2010, Tanzania ranks below the average health professional population density for Africa [8, 9] and far below the WHO recommendation of 1 physician per 1,000 population. There is a little doubt that this health workforce shortage has constituted a key barrier to achieving MDGs 4 and 5 in Tanzania where 1 in 9 children die before the age of five [5, 10], maternal mortality is 432 per 100,000 live births [11, 12], and shortages of medicines and supplies, inadequate infrastructure and poor patient transportation systems amplify the effects of manpower shortage [13].

Several influential observers have argued that this manpower shortage can be addressed by tackling existing inefficiencies in the health system, such as improve limited capacity for planning, deploying and monitoring health staff and utilizing community health workers (CHW) to provide primary health care services [14–16]. In particular, the development of CHWs can accelerate health services utilization and improve the overall effectiveness of primary health care [14, 17] as they are promoting and sensitizing the community on the importance of seeking health care at health facilities. Bhutta and his colleagues [18, 19] and the One Million CHWs Campaign [18, 19] demonstrated and advocated that the deployment of well trained, supplied, supervised and paid CHWs can contribute significantly to improvements of maternal, newborn, and child health (MNCH) as well as survival.

Despite a number of developments and interventions from various development partners in deploying voluntary HW in rural communities, Tanzania continues to faces the challenge of integrating an appropriately trained cadre as part of the national primary health care system [20]. Considerable evidence has shown that home visitation contributes significantly to improving population health, particularly in settings with extreme shortages of capable health professionals [1, 21, 22]. The quality of care and sustainability of CHW programs were generally found to be poor due to lack of continuing professional development and insufficient supervision [23, 24]. However, all CHW programs relied upon unpaid volunteers, most of whom had limited training and specific disease focused roles [25].

### Rationale for training and deploying a new cadre of CHW

The Tanzania Ministry of Health and Social Welfare (MoHSW) instituted the Primary Health Services Development Programme 2007–2017 (*MMAM – Swahili acronym*) to address the manpower shortage. The *MMAM* programme aims to strengthen interactions between health facilities and communities by having a cadre of HW based at a community level [26]. Through *MMAM*, the MoHSW set an objective to reduce the maternal mortality ratio from 578 maternal deaths per 100,000 live births in 2000 to 220 per 100,000 in 2012, and improve newborn care. Nevertheless, by 2012, mortality had only declined to 432 per 100,000 live births [12]. Among the strategies included in *MMAM* was the deployment of CHWs to every village [12, 27]. Although this strategy is in the process of been implemented nationally, it is currently in the stages of training CHWs in a number of public and private institutions. The CHW policy has already been developed and was signed in 2014.

Evidence from CHW programs emphasize community ownership in selecting these workers from the communities they will serve to maintain retention, acceptability and environment awareness [28]. Yet despite efforts in developing strategies for recruitment and deployment of CHWs, there is little information on the full cost of training, deploying and running full-time, paid CHWs on a wide scale. Specifically, most of the CHWs that have been deployed in Tanzania received short term training and contracts for specific short-term interventions [29].

Based on the above concerns, the Ifakara Health Institute (IHI), the Tanzanian MoHSW, the Tanzanian Training Center for International Health (TTCIH) and Columbia University’s Mailman School of Public Health (CU-MSPH) launched in 2011 a CHW program known as *Connect* to evaluate the childhood survival impact of introducing a cadre of paid CHWs known as “Community Health Agents,” which translates into Swahili as “*Wawezashaji wa Afya ya Jamii*” (*WAJA*) into local health systems. The aim of this paper is to estimate the annual unit cost for pre service training, deployment, and maintaining paid CHWs in Tanzania so as to enable policymakers to determine the financial implications of integrating this cadre into the national primary health care system [30].

## Method

### Project design and approach

Connect operates as a randomized cluster trial (RCT) allocating 50 villages to intervention (villages receiving WAJA) and 51 to comparison (villages not receiving WAJA). All 101 villages were located in Kilombero, Ulanga and Rufiji districts of Tanzania where the IHI Health and Demographic Surveillance Systems (HDSS) monitored population dynamics since 1996 to allow for an evaluation of the project’s impact on mortality [31, 32]. Further, the *Connect* economic evaluation aims to assist the MoHSW to determine and model the cost of recruitment, training and deployment of *WAJA*s as envisioned in the *MMAM* [27] and corresponding ongoing policy development.

By August 2012, 113 *WAJA* were trained and deployed in the 50 villages of intervention. Training and deployment were pursued in two phases, with *WAJA* deployed to villages in August 2011 (Batch one = 57 *WAJA*) and August 2012 (Batch two = 56 *WAJA*). Analysis included two phases of *WAJA*s deployment. The project provides funds to districts for human resources, and supplies are directly provided by the project. *WAJA*s link communities to the health system by providing basic curative and preventive health services, health education, promotion and referral to the health care facilities [32].

### Recruitment of WAJA

Recruitment of *WAJA*s involved community sensitization that informed the community about the project and necessary qualifications for selected individuals for *WAJA* training. Selection criteria included residence in the community, at least four years of secondary education, and minimum pass in at least two subjects. Other recruitment activities included posting advertisements to notice boards and other important places in communities. Applicants sent their applications to the village executive officer. With the help of the district education office, the village committee screened applicants’ certificates. At a subsequent meeting, qualified candidates were asked to brief community members about their suitability for the position, after which community members voted to select the *WAJA*(s) for their area. One to four *WAJA*s were selected from each community, based on village population size.

### Training design and process

The *WAJA*s training was held at the TTCIH and all students from Rufiji, Ulanga and Kilombero settled at the training center in Ifakara town. The training spanned nine months and was divided into two sections. The first involved theoretical training that included basic clinical skills, community health skills such as communication and advocacy, counseling, disease prevention, health education, nutrition, primary health care and human biology. A field practice component was also incorporated in the first section of the training, conducted in the villages surrounding the training institution with support from *WAJA* trainers. The second training section was a field attachment, done in the *WAJA*s’ home villages. This involved practicing their skills under the close supervision of trainers and a clinical officer from the nearest health facility. *WAJAs* were then invited to the TTCIH for a final writing and oral examination to test competencies gained during the training. The TTCIH incurred a number of preliminary costs when preparing to accommodate the training such as the renovation of dormitories, hiring a new building for dormitories outside the school, developing curriculum and capacity building for trainers.

### WAJA’s deployment

Deployment proceeded immediately after *WAJA* graduated and passed their final examination. Community members organized a village meeting where the project presented a fully trained *WAJA* with the necessary equipment (bicycle, mobile phone, weighing machines, flip charts, and malaria rapid diagnostic test kits) and medicines (Amoxycillin, Artemeter Lumefantrine (ALu) as an Artemesinin-based Combination Therapy (ACT) known as Coartem, oral rehydration salts (ORS), Zinc oxide plaster etc.). Communities provided a cupboard for storing *WAJA* medicines and supplies. The district health administration was responsible for providing *WAJA* with an employment appointment letter and administering all human resource matters, including *WAJA* salaries (channeled to the district by the project, but paid by district authorities) and social security benefits. *WAJA*s were supervised by intervention coordinators from the project and by the district health administration. A selected HW from a nearby health facility provided technical advice and a village executive officer oversaw *WAJA* activities at the community level. These supervisors met with *WAJA* at least once every three months. *WAJA* phones were also connected to close user group service (CUG), allowing free calls between them and their supervisors for immediate support.

### Data sources

Data on training costs were obtained from the actual expenditures related to schooling costs at the TTCIH. These were extracted from expenditure accounts and improved by in-depth interviews with TTCIH management to provide necessary clarifications. All information was confirmed by records review and interviews of relevant TTCIH and *Connect* project staff. Data on the cost of deployment and annual operation costs of *WAJA*s were extracted from the *Connect* project records. Information was gathered from expenditure records of *Connect* interventions and the actual cost incurred for specific activities like training, salary, supervision, medicines and supplies. The number of *WAJA*s per village and village population size were obtained from project records. Resources expended for on year of *WAJA* operations were tracked from the expenditure accounts and records of quantities of items supplied directly to the *WAJAs*. The national population and village data were drawn from the Tanzania 2012 Census.

The relative training costs of comparable MoHSW operations were gathered from the Clinical Officer Training Centre of Kilosa in Morogoro, a public institution that specializes in two-year training certificates for Assistant Clinical Officers, and three-year diplomas for the lowest level paramedics, Clinical Officers, who provide preventive and treatment services at primary health care facilities. Public Clinical Officer Training Centres were selected due to their future responsibility in scaling up the *WAJA* training program should it be adopted as policy. In-depth interviews with management and financial officers were conducted at the Clinical Officer Training Centre. These, in combination with records review, helped determine the costs of all pre-service training, logistics and operations associated with certificate and/or diploma procedures for one academic year. Information from the Kilosa Training Centre in Morogoro Region, the public institution that trains the lowest level of paramedics, was similarly gathered. The training costs of Assistant Clinical Officers were ultimately used as a benchmark, as this cadre is most similarly comparable to the education level required for the *WAJA*s.

### Data analysis

Analysis involved valuing each resources used in every activity related to *WAJA* training, deployment and annual running costs. Interview responses were coded and grouped into specific groups to clarify mentioned categories. Actual costs from the records were then combined based on this information.

The unit training cost of *WAJA* was calculated as the total cost of establishing *WAJA*s divided by the number of *WAJA*s trained by the project. The unit training cost of Assistant Clinical Officers was calculated as the annual cost of training divided by the number of trainees.

Modeling scale-up training cost using project expenditure and clinical training centre information followed, and was developed according to seven assumptions :i) The training of *WAJA* will be undertaken by existing government training institutions and will be within their existing competence; ii) Tuition allowance, training facilities, and utilities are to be included into an overall tuition fee; iii) Curriculum and identity card costs are included in tuition fees; iv) Meals are provided for students undergoing training; v) Transport costs for *WAJA* to travel to training and return to their villages are not included in the estimates; vi) Students will have two months of field attachment; and vii) The district health system is the unit of planning. After deploying *WAJA*s to the health system at the village level, resources to run the program for a full year were tracked and related costs recorded to determine annual running costs for the program.

Our estimation was based on the number of *WAJA*s required to serve: 1) A standard-sized district in Tanzania; and 2) The entire country. This estimate was based on the population of the villages as assessed in *Connect* project. Villages with populations less than 1,000 people received 1 *WAJA*; villages with a population of 1000 to 4000 received 2 *WAJAs*; villages with a population of 4000 to 7000 received 3 *WAJAs*; and those with more than 7000 received 4 *WAJAs*. To determine the standard size of a district, villages in project districts were summed and averaged to have a mean number of villages in a “standard-sized district”.

The trial is registered with the International Standard Randomized Controlled Trial Register (number ISRCTN96819844).

## Results

Table 1 presents various program activities related to *WAJA* training and the unit cost per *WAJA* trained. Costs are presented in the 2011 equivalent US dollar (US$) value of the local currency, Tanzanian Shilling (TSH)^1^ at the time of deployment.

**Table 1:** Unit cost for training a community health agent (*WAJA*), *Connect* Project, 2011

Cost category	Unit cost (USD) Per *WAJA* deployed	Percentage (%)
Meals	997.31	40.1
Accommodation	502.97	20.2
Tuition fee	254.21	10.2
*WAJA* stipend	199.37	8.0
Training facility and utilities	195.04	7.8
Field allowance	101.99	4.1
Medical insurance	23.86	1.0
Curriculum development	2.34	0.1
Other	212.16	8.5
Total Unit Training Cost	2,489.25	100.0

Source: Connect project WAJA training data

The unit cost of the training program was US$ 2,489.3 per trainee with the highest percentage (40.1 %) spent on meals. Accommodation during training accounted for 20.2 %, tuition fees were 10.2 %, and stipends, field allowances and training facilities and utilities accounted for 19.9 % of the total unit training costs. Other costs accounted for 8.5 %, and medical insurance for trainees comprised 1.0 % of the total unit cost.

Table 2 shows the equivalent cost of one year of training for an Assistant Clinical Officer in 2011 at the Kilosa Training Centre. The annual unit training cost for an Assistant Clinical Officer was US$ 731.0, where meals accounted for the highest portion of the total training cost at 55.1 %. The field attachment accounted for 15.6 % of the total cost, followed by tuition fees, accommodation, and utilities at 15.3, 8.5, and 5.1 %, respectively. Stationery comprised less than 1 % of the total training cost.

**Table 2:** Unit cost for training an assistant clinical officer at a public training centre in Tanzania in 2011

Cost category	Unit cost (USD) Per *WAJA* deployed	Percentage (%)
Meals	403.0	55.1
Field allowance	114.0	15.6
Tuition fee	112.0	15.3
Accommodation	62.0	8.5
Utility	37.0	5.1
Stationary	4.0	0.5
Total Unit Training Cost	731	100.0

Source: Public clinical officer training centre, Kilosa-Morogoro

As shown in Table 3, the modeled scaling up cost to train a *WAJA* based on the Kilosa Public Clinical Officer Training Centre is estimated at US$ 833.50. Half of this cost (50.3 %) would involve meals, 27.4 % tuition fees, 13.7 % field allowance, 6.0 % accommodation and 2.7 % medical insurance.

**Table 3:** Modeled training cost based on *Connect* project and clinical officer training centre

Cost Category	Unit cost (USD) Per *WAJA* deployed	Percentage (%)
Meals	419.19	50.3
Tuition fee	228.40	27.4
Field allowance	113.98	13.7
Accommodation	49.67	6.0
Medical insurance	22.25	2.7
Modeled Unit Training Cost *WAJA*	833.49	100.0

Source: Calculated by the authors based on scenario of project expenditure and public clinical officer training centre

Table 4 presents the annual running cost for the 113 *WAJA*s deployed to the *Connect* intervention districts, totaling US$ 407,954 per year. The highest portion of costs were associated with *WAJA* salaries at 48.3 %, followed by drugs/medicines at 35.3 %, supporting structures at 14.2 % and medical supplies at 2.3 %. The cost of operating one *WAJA* in a village is US$ 3,610, which is equal to US$ 1.16 per capita per year.

**Table 4:** Annual cost of running Connect

Cost category	Cost (USD)	Percentage
*Project costs of program implementation:*		
Support structure	57,794	14.2
Salary	196,839	48.3
Medicine	144,020	35.3
Supplies	9,301	2.3
Total financial cost	407,954	100.0
*Estimated unit costs of program implementation:*		
Unit annual cost per *WAJA*	3,610	
Per capita cost	1.16	

Source: Connect project running/operation data

To scale up *WAJA* training to a standard district, based on *Connect* allocation, covering all villages was estimated to cost US$ 180,206, which covered training of approximately 216 *WAJA*. The training to cover the entire country will require training for 27,120 *WAJA*s. Training is estimated to cost US$ 0.5 per capita and can be implemented by zones in different batches for a number of years.

## Discussion

We have estimated the unit cost of comprehensive training, deployment and annual running cost of a paid *WAJA*. The analysis estimates these costs in the current health system structure, and, thereby, costs that offer a potential solution to the shortage of primary health care human resources [33] in Tanzania. Programs elsewhere have recruited newly-graduated students of various levels and trained them for various durations to become CHWs. For example, in India such workers were trained for three months, whereas in Brazil, they were trained for six to eight months prior to their deployment [34]. However, for most such programs, as in Uganda, Ethiopia and Rwanda, the cost of training, deployment and running a CHW remains unexhausted as it has not captured the full package of CHW tasks [17, 23]. However, few studies have pointed out a shadow value for CHW as they work in informal market [35].

This study has estimated the cost of scaling up *WAJA* training at public training centres to be US$ 833.50 per *WAJA*, compared with the *Connect* project actual training cost of US$ 2,489 per *WAJA*. The *Connect* project training was done in an institution that runs in-service training and technical training such as advanced diplomas, degrees and post-graduate diplomas. The scaling up cost is less and provides best proxy as it is derived from the cost of training clinical officers at regional and district training centres. The training profile of these institutions reflects the cost of training primary health care staff who are hired at the minimum education level of four years of secondary education and basic technical education. The running of such training exists at a number of regional training institutions mostly found at regional hospitals and few district hospitals. The training of such workers resembles *WAJA* training, where the cost of meals, teaching methodology and supporting staff is similar, except its training duration, which is twice as long as that of *WAJA*. A high proportion of training costs was comprised of meals and accommodation at 57 and 60 % of training costs of the estimated scaling up Assistance clinical officers and *WAJA*.

The difference between estimated *Connect* Project costs and estimated program scale-up costs is based on the utilization of existing data on the operating costs of public clinical officer training centres, given prevailing residency, training, and meal expenses. Project expenses of *Connect WAJA* training involved accommodating students in hired dormitories outside the campus, while at the clinical officer training centre, students stayed in less costly arrangements at dormitories located inside the campus. Thus, if scale-up is based solely in clinical officer training centres under National council for technical education, the implementation costs of *WAJA* scale-up will be reduced, particularly if existing public college training facilities accommodate students and provide meals. However, estimating the full cost of public sector student training is complicated due to the presence of undocumented subsidies that arise from the government and the community to support the institution, such as security and community contributions to building or renovating new infrastructures [36]. Although the *WAJA* program has incurred far less indirect support than has been the case for the implementation of other HW cadres, some costs can be substantial and can lead to variance in overall implementation costs. For example, Mullan and Frehywot found that the annual cost for training non-physicians in Tanzania varies considerably from one non-physician cadre to another, with costs ranging from US$ 1,300 to US$ 2,000 [36].

Sustainable training of *WAJA*s can be conducted by selecting training facilities at zonal, regional or district levels [37] such as the Clinical officer training centre, Kilosa. These institutions can be responsible for evaluating the need for training, conducting recruitment, and providing training [37]. This will reduce training problems that arise as a result of fragmented, piecemeal, or different training facilities that may lead to poor quality of training if they are not amenable to large training groups [38].

Currently, policy makers primarily focus on the training of doctors, nurses, and other mid-level health personnel [39]. This study may help policymakers allocate resources for training lower cadres of HW such as *WAJAs* to solve the existing human resource limitations in providing access to health services. The number of HW can be increased by promoting the WHO approach of recruiting and training local people, who will then reside and provide services in their respective localities.

The government should lead the scale- up and sustainability for a cadre of CHW in Tanzania using its existing health systems, training and administrative structures. This will have the potential to require fewer resources, as the estimated cost is less than that of non-government programmes seeking to achieve the same goal [40]. According to this model, the relative cost and time required to train, deploy and run advanced skilled cadres per year [36], compared to CHW, the CHWs has greater potential to be scaled-up from an economic perspective compared to other dispensing cadres to be deployed to the villages.

Our findings are comparable to results reported in a study on rural sub-Saharan Africa by McCord on 2013. These results showed that CHW average annual deployment costs were approximately US$3,750 for rural sub-Saharan Africa, compared to US$3,610 for *WAJA.* CHW average annual deployment cost per capita was US$2.72 for rural sub-Saharan Africa compared to US$1.16 for *WAJA* [40]. This can be manageable at the community level, if the health manager at the districts level will prioritize this and sensitize to community ownership.

### Limitations

Training curriculum development was deemed to be a once only project activity that would contribute to national training plans. The analysis bounded to two cohorts of training, covering a total of 113 *WAJA*. For this reason, the study did not take into account the incremental salary and benefits associated with the contribution of the lead trainer and the training supporting staff that developed the curriculum. Also, the infrastructure associated with training sessions has not been included in costing. *WAJA* family additional support costs for incidental spending and clothing are not factored into estimates of incremental *WAJA* costs. Lastly, the scaling up analysis did not account for the attrition rate, this can be one of the factors hindering the sustainability of the program at large scale.

## Conclusion

To sustainably train, deploy and run a cadre of community-based health personnel, the government must take into consideration the factors that may hinder the continuous production of HWs according to demand for health care at the community level. These factors include whether the number of HWs being trained and deployed will be sufficient to meet the demand for these health personnel, and whether there is a need to build new schools or expand existing training facilities to meet expected requirements.

## Abbreviation

CHW, Community Health Workers; HDSS, Health and Demographic Surveillance Systems; HW, Health Workers; IHI, Ifakara Health Institute; MDGs, Millennium Development Goals; MNCH, Maternal, Newborn, and Child Health; MoHSW, Ministry of Health and Social Welfare; TTCIH, Tanzanian Training Centre for International Health; WAJA, “Wawazesha wa afya ya Jamii”; WHO, World Health Organization

## Footnotes

1 Bank of Tanzania 2011 report (1 USD = TSH 1510).
